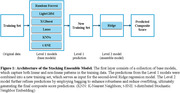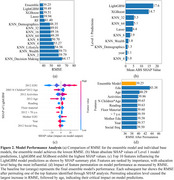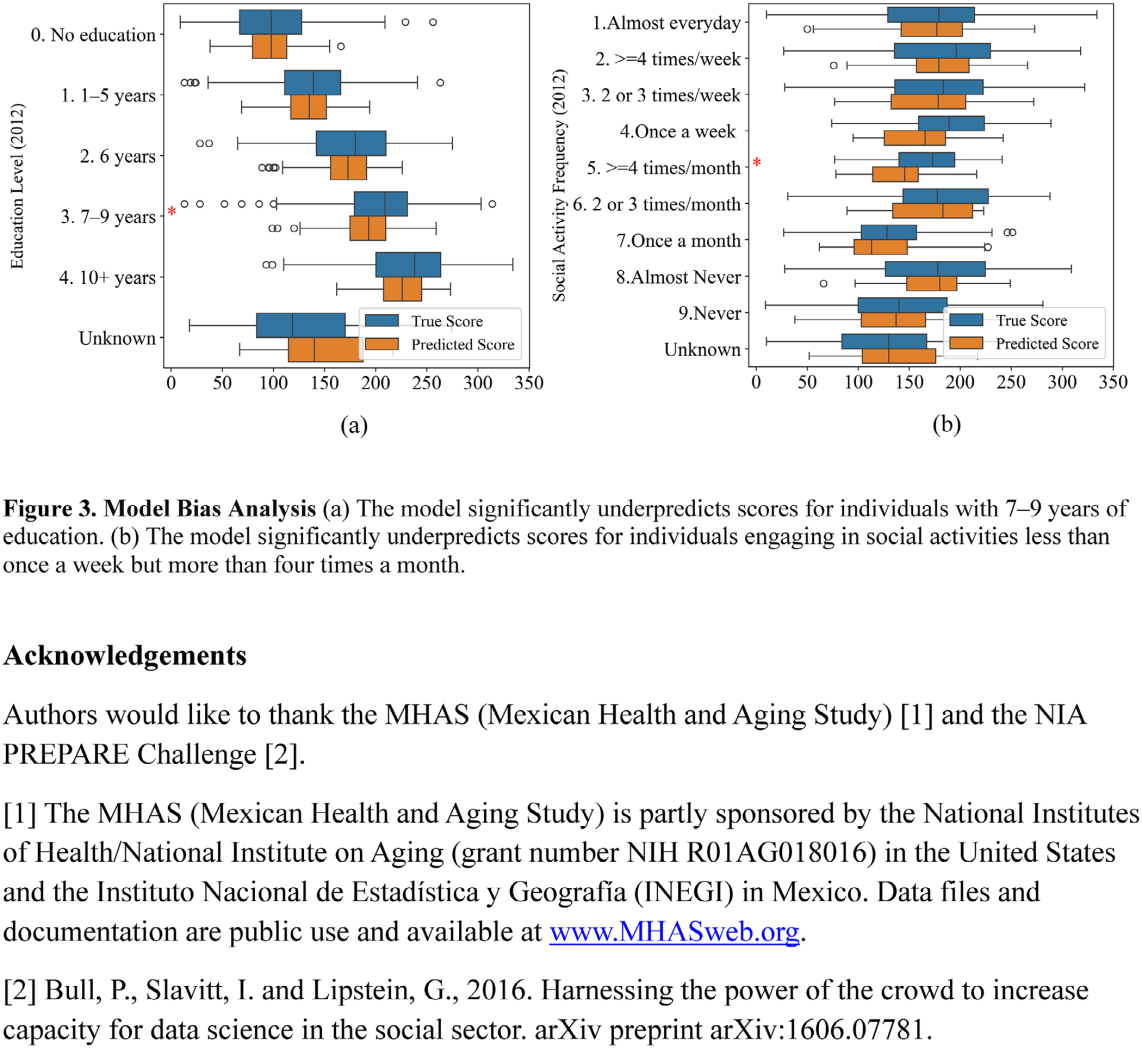# Early Detection of Cognitive Impairment Using Social Determinants of Health

**DOI:** 10.1002/alz70857_107667

**Published:** 2025-12-26

**Authors:** Yignan He, Yu Leng, Ana‐Maria Vranceanu, Christine S Ritchie, M. Maria Glymour, Deborah Blacker, Sudeshna Das

**Affiliations:** ^1^ Massachusetts General Hospital, Boston, MA, USA; ^2^ Massachusetts General Hospital (MGH), Boston, MA, USA; ^3^ Boston University School of Public Health, Boston, MA, USA

## Abstract

**Background:**

Early diagnosis of Alzheimer's disease and related dementias (AD/ADRD) is essential for timely intervention, but many current screening methods, such as blood‐based biomarkers and imaging techniques, remain inaccessible. Social determinants of health (SDoH) and lifestyle factors offer readily available alternative approach to predicting cognitive decline. Thus, we developed a stacking ensemble model that uses SDoH data for early detection of cognitive impairment.

**Method:**

Our model was trained using data from the Mexican Health and Aging Study (MHAS), a national longitudinal survey of adults aged 50 and older in Mexico (*n* = 4,095). The dataset includes variables covering demographics, economic conditions, self‐reported health, and lifestyle behaviors collected in 2003 and 2012. The model employs a two‐level stacking ensemble architecture to predict global cognition in 2016 and 2021 (Figure 1) (1) First‐layer models provide feature representations; (2) Second‐layer model integrates predictions from the first‐layer models to predict the cognitive composite score.

**Result:**

The ensemble model achieved a Root‐Mean‐Square Deviation (RMSE) of 39.25 on the test set (Figure 2a), corresponding to an average deviation of approximately 10.2% from the full range of the cognitive score (0 to 384). Among the base models, LightGBM demonstrated the best performance (Figure 2b). Feature importance analysis highlighted the key social and lifestyle factors influencing cognitive function (Figure 2c). Sensitivity analyses showed that perturbations to top‐ranked features caused minimal RMSE fluctuations, demonstrating model robustness (Figure 2d). Additionally, model performance analysis revealed biases in certain education and social engagement subgroups, potentially because of lower representation of those subgroups in the dataset (Figure 3).

**Conclusion:**

Our study demonstrates the feasibility of using readily available SDOH data for early detection of cognitive impairment. The model provides a balance between predictive performance and explainability. Future work should predict change in cognitive scores since cognitive scores are influenced by social determinants of health.